# Source diversity of *Artemia* enrichment boosts goldfish (*Carassius auratus*) performance, β-carotene content, pigmentation, immune-physiological and transcriptomic responses

**DOI:** 10.1038/s41598-023-48621-4

**Published:** 2023-12-09

**Authors:** Ahmed E. Elshafey, Malik M. Khalafalla, Attia A. Abou Zaid, Radi A. Mohamed, Mohamed M. Abdel-Rahim

**Affiliations:** 1 Department of Aquaculture, Faculty of Aquatic and Fisheries Sciences, Kafrelsheikh University, Kafrelsheikh, 33516 Egypt; 2 National Institute of Oceanography and Fisheries (NIOF), Cairo, 21556 Egypt

**Keywords:** Developmental biology, Ecology, Immunology

## Abstract

This study aimed to assess the impact of spirulina and/or canthaxanthin-enriched *Artemia* on the goldfish (*Carassius auratus*) growth, pigmentation, blood analysis, immunity, intestine and liver histomorphology, and expression of somatolactin (SL) and growth hormone (GH) genes. *Artemia* was enriched with spirulina and/or canthaxanthin for 24 h. Goldfish (N = 225, 1.10 ± 0.02 g) were tested in five experimental treatments, three replicates each: (T1) fish fed a commercial diet; (T2) fish fed a commercial diet and un-enriched *Artemia* (UEA); (T3) fish fed a commercial diet and spirulina-enriched *Artemia* (SEA); (T4) fish fed a commercial diet and canthaxanthin-enriched *Artemia* (CEA); and (T5) fish fed a commercial diet and spirulina and canthaxanthin-enriched *Artemia* (SCA) for 90 days. The results showed that the use of spirulina and/or canthaxanthin increased performance, β-carotene content and polyunsaturated fatty acids of *Artemia*. For goldfish, T5 showed the highest growth performance, β-carotene concentration and the lowest chromatic deformity. T5 also showed improved hematology profile, serum biochemical, and immunological parameters. Histomorphology of the intestine revealed an increase in villi length and goblet cells number in the anterior and middle intestine, with normal liver structure in T5. SL and GH gene expression in the liver and brain differed significantly among treatments with a significant increase in enriched *Artemia* treatments compared to T1 and T2. In conclusion, the use of spirulina and/or canthaxanthin improved performance of *Artemia.* Feeding goldfish spirulina and/or canthaxanthin-enriched *Artemia* improved performance, β-carotene content, pigmentation, health status and immune-physiological response.

## Introduction

Ornamental fishkeeping is a popular interest for both young and old. It contributes to human well-being by teaching children responsibility, reducing adult stress, and assisting elderly with essential physical and psychological difficulties^1^. One of the most famous and highly valued ornamental fish is the goldfish, *Carassius auratus* that belongs to the Cyprinidae family^2,3^. Goldfish became very popular and numerous farms and hatcheries are now scattered in the Delta region of north Egypt^4^. Due to its natural beauty and its ability to grow in a diverse range of environmental conditions, goldfish became extremely desirable commercially among aquarium keepers^3,5^. Moreover, goldfish are widely raised as aesthetic pets^6^. The goldfish is a popular tank species and a suitable laboratory animal^7^. Domestication and artificial breeding of goldfish may be dated back thousands of years in Asia^8^.

Goldfish is characterized by an attractive red color which originates from high levels of carotenoids in the fish tissue and this color is responsible for customer acceptability and competitive price^9,10^. In teleost’s, six chromatophores have been identified: cyanophores, leucophores, iridophores, xanthophores, and melanophores^11,12^ stated that Color variations occur at different locations on the fish body due to the distribution of distinct chromatophores. The pigment cell types in this species' dark and bright regions are different. Carotenoids are also essential for growth, metabolism, reproduction, and pigmentation of goldfish^13^. Like other animals, fish are incapable of synthesizing carotenoids^14^, thus, they must obtain them from their food. The pigmentation of fish is affected by the consumption of carotenoids because of this, numerous sources of carotenoid colors, including pure carotenoid pigments, animal-derived pigments, and plant-derived pigments, have been added to fish diets^15,16^. In fish feed, plant sources provide a double benefit: direct access to nutrients such as protein, fats, and vitamins, and a high carotenoid content^17^.

Recently, plant elements like leaves, fruit peels, and flowers are used to enhance the colors of ornamental fish. A nutritionally balanced diet with all the basic nutrients and carotenoids dietary supplements is necessary for intensive ornamental fish production^18^. Nutritional specifications in the feed are among the most critical aspects of goldfish rearing because they cannot synthesize Eicosapentaenoic acid (EPA) and Docosahexaenoic (DHA) acid from shorter polyunsaturated fatty acids (PUFA), which are necessary for goldfish growth and survival^10^. Current efforts focus on natural carotenoids as a possible replacement for synthetic carotenoids due to concerns regarding synthetic additives and their high price^19^, for instance, spirulina (blue green algae) as a natural source of carotenoids^20,21^.

Live food organisms consist of several types of phytoplankton and zooplankton and mostly, zooplankton eats phytoplankton therefore phytoplankton is the basis of the food chain^22^ that plays a great role in nutrition of many fish and crustaceans in their larval stages^19,20^. It is essential for the survival and development of fish larvae because they provide a source of glucose, fat, and protein^10^. Live foods are more likely to promote larval feeding due to their swimming patterns in the water column, their availability to fish and shellfish larvae, and their ability to be easily identified and captured^23–25^. During the fish larval rearing phases, live foods such as *Artemia* sp., *Daphnia* sp., *Tubifex* sp., and *Moina* sp. are routinely used^26,27^. It is hypothesized that the mobility, metabolic wastes, and chemical attractants of living food organisms facilitate fish larval feeding^27–29^. In addition, live food organisms are more digestible than artificial micro diets, and they enhance digestion by providing exogenous enzymes^27,30^.

*Artemia* nauplii are the most common live food used for rearing goldfish larvae. Reference^31^ revealed that the speedy feeding response of *Artemia* is triggered by visual and chemical signals, including movement and the production of chemical attractants (free amino acids, betaine, and peptides). After its entry into the fish larval intestine, *Artemia* nauplii may play a crucial function in the process of feed digestion^32^. *Artemia*'s with its nonselective feeding behavior makes it an ideal biological carrier for bioencapsulating (live food enrichment) critical nutrients that improves survival, development, and quality of aquatic larvae^33^.

Furthermore, spirulina is used as a prominent feed additive for fish^34,35^. Spirulina is rich with bioactive substances such as chlorophyll, carotenoids, and phycobiliproteins which are colored substances that might be used as food pigments^35^. Despite the fact that spirulina-derived pigments are less stable than their synthetic ones, they may give extra health advantages upon ingestion, since carotenoids contain provitamin A and their consumption improves immune response of fish^34,36^. Apart from being high in protein, microalgae also contain a lot of vitamins, fat (particularly polyunsaturated fatty acids, or PUFAs), which are good for aquatic animals' growth, reproduction, and immunity, a lot of polysaccharides, which can boost an animal's immunity, and a lot of pigments, like lutein, astaxanthin, and β-carotene, which can enhance an animal's color^37^.

Canthaxanthin (C_40_H_52_O_2_) was discovered in the edible mushroom *Cantharellus cinnabarinus*^38^. Even though they exist naturally, they are found in a broad range of fish (golden mullet, carp, wrasse, and seabreams), crustaceans, bacteria, and certain green algae^39^. Canthaxanthin was identified as a byproduct of the conversion of carotene to astaxanthin^38,40^.

One of the most noticeable phenotypic traits of fish is body color, which is crucial for courting, immunity, concealment, and predation. The distribution and combinations of several pigment cell types determine how body color is formed^41^. Based on the mechanics behind color production, body color can be classified into two categories: pigment color and structural color. Fish color is ultimately determined by the mixture of these two pigment patterns^12^. Due to the fact that the color fading of goldfish negatively impacts the success of ornamental fish farmers and pet stores, the aim of this study was therefore to evaluate the degree to which goldfish larvae fed live *Artemia* enriched with spirulina and/or canthaxanthin differ in terms of growth performance, pigmentation, blood analysis, intestine and liver histomorphology and gene expression. A second aim of the current study was to evaluate the performance, β-carotene concentration, and fatty acids content of *Artemia* supplemented with spirulina and/or canthaxanthin.

## Materials and methods

### Ethical approval

The current study was approved and methods were performed in accordance with the relevant guidelines and regulations of the Institutional Committee of Aquatic Animal Care and Use in research, Faculty of Aquatic and Fisheries Sciences, Kafrelsheikh University (approval number: IAACUC-KSU-5-2021).

### Preparation of un-enriched and enriched *Artemia* nauplii

The brine shrimp (*Artemia salina*) eggs were purchased commercially from a brine shrimp (El-Max research station, Alexandria, Egypt). *Artemia* cysts weighing 0.004 g (250,000 cysts g^−1^) were incubated in 1 L of salted water (30–33 ppt) in a plastic bottle with a conical bottom that was mildly aerated (constant oxygen supply) and illuminated with 2000 lx during the hatching process. The water used for hatching and enriching *Artemia* had a pH of 8.13, total dissolved salts (TDS) of 4.22 g L^−1^, and a temperature of 28 °C^42^ and *Artemia* hatched 24 h later. Cyst shells float to the surface at the same time. For 24 h, a large portion of *Artemia* was enriched with three carotenoid sources: spirulina (0.5 g L^−1^) (SEA), canthaxanthin (0.5 g L^−1^) (CEA), and a combination of both spirulina and canthaxanthin (0.5 and 0.5 g L^−1^ respectively) (SCA) representing T3, T4, and T5 respectively, whereas T2 was fed un-enriched *Artemia* (UEA) and T1 was fed a commercial diet only according to^43^. Spirulina and canthaxanthin were dissolved in the water and mixed thoroughly with the water in the plastic bottle with a conical bottom. To collect enriched *Artemia*, the aeration was removed to allow the nauplii to gather at the conical portion of the plastic bottle. Enriched *Artemia* was sucked from the plastic bottle after five minutes, collected on fine gauze, and washed with distilled water to remove any adherent food particles. *Artemia* was dried and weighed on a filter paper. Enriched *Artemia* was produced on a daily basis in order to provide goldfish with enriched *Artemia* on a constant basis.

### Goldfish and rearing systems

The goldfish fry (N = 400, 1.10 ± 0.02 g) were obtained from a private commercial hatchery and transported in polyethylene plastic bags (1 water:2 pure oxygen) to the glass aquarium laboratory. Tap water was used in this experiment and aerated for 72 h to ensure that there is no residual chlorine content. Water temperature was 28.45 °C, pH was 7.20, dissolved oxygen was > 6 mg L^−1^, and unionized ammonia was less than 0.001 mg L^−1^. The water was filtered through a 0.45 μm membrane filter prior to use. The goldfish were acclimated for two weeks before the start of the trial. The proximate composition of the commercial diet used during the adaptation period contained 90% dry matter (DM), 44% crude protein, 9% ether extract, 7% ash, 36.62% Nitrogen-free extract (NFE), 3.38% crude fiber and 4520 kcal kg^−1^ gross energy. After the acclimatization period, the fish (n = 225) were distributed into 15 glass aquariums (45 cm × 30 cm × 25 cm and 30 L capacity) representing five experimental treatments and three replicate each (15 fish per aquarium). Each aquarium was supported with an aeration system from a radial compressor with fine-porous stones placed at the bottom of each aquarium. The experimental units were placed in a wet laboratory with artificial lighting and a controlled photoperiod (12 light:12 dark) and the experiment lasted for 90 days.

### Experimental treatments

**Figure 1:**
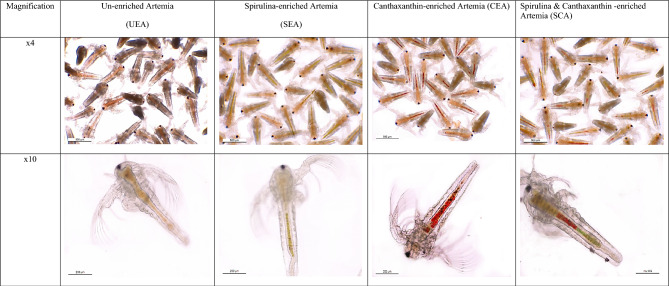
Un-enriched and enriched *Artemia* with spirulina and/or canthaxanthin; imaged using a Leica microscope (LEICA ICC50W).

The experimental fish were randomly allocated into five treatments as follows: treatment one (T1): goldfish fed a commercial diet (10% of body mass, adjusted according to fish till reach 5% at the end of 4th week then continue with 5% till the end of 90 days experimental period); treatment two (T2): fish fed commercial diet and un-enriched *Artemia* at 200 pieces per goldfish/day^44–46^; treatment three (T3): fish fed commercial diet and spirulina-enriched *Artemia* at 200 pieces per goldfish/day; treatment four (T4): fish fed commercial diet and canthaxanthin-enriched *Artemia* at 200 pieces per goldfish/day; and treatment five (T5): fish fed commercial diet and *Artemia* enriched with both spirulina and canthaxanthin at 200 pieces per goldfish/day. The goldfish received three equal meals (9 a.m., 12 p.m., and 3 p.m.) per day containing commercial diet for T1 only. For T2, T3, T4, and T5, fish received one meal (at 9 a.m.) containing a commercial diet of the same amount introduced to fish in T1 and two *Artemia* meals (at 12 p.m., and 3 p.m.). The success of the enriched process is illustrated in (Fig. 1). Images of un-enriched and enriched *Artemia* with spirulina, canthaxanthin, or both were taken with a Leica microscope (LEICA ICC50W) (www.leica-microsystems.com). The approximate composition of the commercial diet contained 90% dry matter (DM), 44% crude protein, 9% ether extract, 7% ash, 36.62% Nitrogen-free extract (NFE), 3.38% crude fiber and 4520 kcal kg^−1^ gross energy. Unconsumed feed was collected daily, and feces were removed and 25% of the water was refreshed every 2 days.

### Experimental measurments

#### Artemia

##### Proximate composition and characteristics of *Artemia*

Proximate composition analysis of moisture, crude protein, crude lipid, fiber, and ash of experimental finished diets were determined according to analytical procedures described in^47^. For each analysis, triplicate samples were used. Every day, the survival rate of each treatment was calculated by observing and calculating the number of *Artemia*. A high-precision microscope (Nikon, Japan) was used to measure the length of each *Artemia*. A 100 mL water sample was taken from each replicate tank using the random sampling technique to determine the density and biomass of *Artemia*. The biomass of *Artemia* was measured on a dry weight basis with a microbalance (Electrical Analytical Balance, OSK 11325A), and the size of *Artemia* was measured isobar on a LEICA ICC50 (www.leica-microsystems.com).

##### Total carotenoid assessment of *Artemia* using UV–VIS spectrophotometer

Total carotenoids content was determined using a spectrophotometer (Specord 210, model Analytikjena) set to 450 nm as the method described by^48^.

##### Fatty acid analysis of *Artemia*

Lipid extraction: Weigh 2–20 g of the sample into a 250 mL centrifuge bottle; add sufficient water to bring total water present to 16 mL together with 40 mL methanol and 20 mL chloroform. Macerate for 2 min; add further 20 mL chloroform and macerate for 30 s; add 20 mL water and macerate again for 30 s. Centrifuge the mixture for 10 min at 2000–2500 rpm. Draw off the lower chloroform layer and filter through a coarse filter paper into a dry weighed flask or beaker. Evaporate the chloroform to dryness^49^. Methylation of lipid in a tube weighs 50 mg of lipid, add 5 mL of methanolic sulphuric acid (1 mL conc. sulphuric acid and 100 mL methanol) and 2 mL of benzene, close the tube well, and place in a water bath at 90 °C for an hour and a half. Cool, add 8 mL water and 5 mL petroleum ether, shake strongly, separate the ethereal layer in a dry tube and evaporate to dryness^50^. Coupling of two-dimensional thin layers chromatography with gas chromatography (GC) were used for the quantitative analysis of lipid classes and their constituent fatty acids.

#### Goldfish studied

##### Water quality analyses

Water quality parameters were measured twice per week in the midpoint of each aquarium. Temperature, pH, dissolved oxygen (DO), electric conductivity (EC) and total dissolved salts (TDS) were analyzed according to established protocols (APHA 1976) using Multi parameters probe meter (HI9829–03042-HANNA®insrruments, www.hannainst.com). Total ammonia nitrogen (TAN) was estimated using a portable photometer (Martini MI 405 MR). According to^51^ unionized ammonia (UIA) was determined from the pre-estimated TAN, temperature, and pH.

##### Growth performance and condition factor of goldfish

For each experimental treatment, 30 fish were randomly selected bi-weekly to estimate the periodical gain in weight and length. At the end of 90 days experimental period, goldfish were anesthetized by clove oil (Merck, Germany, 50 μL L^−1^), collected, counted, and weighed. The growth performance and feed utilization parameters were determined as follows:

$$\eqalign{
   & {\text{Weight gain}}\left( {{\text{g fis}}{{\text{h}}^{ - 1}}} \right),\;{\text{WG}} = {\text{Wt}} - {\text{W}}0;\;{\text{Average daily gain ADG }}\left( {{\text{g fis}}{{\text{h}}^{ - 1}}\,{\text{da}}{{\text{y}}^{ - 1}}} \right) = {\text{Wt}} - {\text{W}}0\,{\text{day}}{{\text{s}}^{ - 1}};\;{\text{Specific growth rate }}\left( {\% {\text{ da}}{{\text{y}}^{ - 1}}} \right); \cr 
   & {\text{SGR}} = 100 \times \left( {\ln \;{\text{Wt}} - \ln \;{\text{W}}0{\text{/experimental period}}} \right),\;{\text{where:}}\;{\text{W}}0{\text{:}}\;{\text{initial fish weight }}\left( {\text{g}} \right),{\text{ Wt: final fish weight }}\left( {\text{g}} \right),{\text{ }}\ln {\text{: natural logarithm}}; \cr 
   & {\text{Survival rate }}\left( \%  \right) = 100 \times ({\text{final number of fish/initial number of fish}}). \cr} $$

$$\eqalign{
  \,{\text{Length gain }}\left( {{\text{cm fis}}{{\text{h}}^{ - 1}}} \right) = {\text{Lt}} - {\text{L}}0;{\text{ condition factor }}\left( {\text{K}} \right) = 100 \times \left( {{\text{W}}1{\text{/L}}{{\text{t}}^3}} \right) \cr 
   & {\text{where}},{\text{ Lt: final fish length }}\left( {{\text{cm}}} \right),{\text{ L}}0{\text{: initial fish length }}\left( {{\text{cm}}} \right). \cr} $$

##### Proximate chemical composition of goldfish

Moisture, crude protein, crude lipid, fiber, and ash composition of experimental goldfish at the end of the experiment were determined using the analytical methodologies described in^47^. Triplicate samples (10 g dry matter/sample) from each experimental treatment were used for each analysis.

##### Analysis of β-carotene in experimental goldfish using high-performance liquid chromatography (HPLC)

The triplicate sample (10 g) was used for detection of β-carotene in experimental goldfish using high-performance liquid chromatography (HPLC) according to^52^. The wavelength of detection was 476 nm.

##### Chromatic deformity and color intensity using computer-assisted image analysis

ImageJ 3 software was used to perform computer-assisted image analysis on photographs (National Institute of Mental Health, Bethesda, Maryland, USA). On the final day of the experiment, 15 randomly selected goldfish per each group (5 fish/replicate) were photographed under standard light conditions (lamp type: Philips TL-D Special 90; two tubes of F95, color temperature: 5300°K; one tube of F33, color temperature: 4100°K) using a digital camera (Olympus C-2000 Zoom; Olympus Optical, Tokyo, Japan). According to the procedures of^53^, grayscale values (0–255) of the R, G, and B components of the RGB scale were measured within a 30×30-pixel area of the red dorsal body surface.

##### Blood sampling and serum separation

The blood samples (six fish/replicate) were collected from the caudal vertebral vein of an anesthetized fish using a syringe (Masco Mid, Tanta city, Egypt) with finer gauge needle to reduce the pain and for easy blood collection according to^54^ at the end of the feeding experiment. The blood collected from each two fish was pooled to form one sample. The collected blood sample was divided into two tubes, one containing EDTA as an anticoagulant agent for hematological analysis. The other tube was without anticoagulant for serum separation. The blood was stored in a refrigerator for 4 h till colt formation then a centrifugation of blood clot was done at 4000 rpm/15 min at 4 °C and stored at − 20 °C until used for further analysis.

Hematological analyses: The erythrocytes and leukocytes were counted according to the method described by^55^ using neubauer hemocytometer and Natt-Herrik solution. Hemoglobin concentration was determined according to^55^ using the cyanomet hemoglobin method Drabkin's solution. According to^56^, the microhematocrit method was conducted to determine the packed cell volume (PCV). In order to determine differential leukocytic count (DLC), thin blood films were prepared, air-dried, fixed with methanol for 3–5 min, stained with Gimsa stain for 8–10 min, and then allowed to dry. The white blood cells were counted among one hundred blood smears according to^55,57^.

Serum biochemical analyses: Serum total proteins were determined colorimetrically at the wavelength of 546 nm using the commercial kits (REF:310 001 Spectrum, Egyptian company for Biotechnology, Egypt)^58^. Albumins were determined colorimetrically at the wavelength of 630 nm using the commercial kits (CAT. No. AB 10 10, Biodiagnostic Co. Egypt)^59^. Globulin content was calculated mathematically (globulin = total protein − albumin). The activities of aspartate aminotransferase (AST) were measured colorimetrically at 505 nm using the commercial kits (CAT. No. AS 10 61 (45), Biodiagnostic Co. Egypt)^60^. Alanine aminotransferase (ALT) activities were measured colorimetrically at 505 nm using the commercial kits (CAT. No. AL 10 31 (45), Biodiagnostic Co. Egypt)^60^. Levels of triglycerides were determined colorimetrically at the wavelength of 505 nm using the commercial kits (CAT. No. TR 20 30, Biodiagnostic co. Egypt)^61^. Cholesterol level was determined colorimetrically at the wavelength of 500 nm using the commercial kits (CAT. No. CH 12 20, Biodiagnostic Co. Egypt)^62,63^. Serum glucose level was assessed using commercially available kits (glucose enzymatic PAP kits, Bio-Merieux, France)^64^.

Digestive enzymes activity: Lipase activity was determined colorimetrically at the wavelength of 580 nm^65^ using commercial kits (REF:281 001 Spectrum, Egyptian company for Biotechnology, Egypt). Amylase activity was determined colorimetrically at the wavelength of 660 nm as the method described by^66^ using commercial kits (CAT. NO. AY 10 50, Biodiagnostic Co. Egypt).

Immunological analysis: The lysozyme activity was assayed by ELISA according to the method described by^67^ based on the ability of lysozyme to lyse Gram-positive lysozyme sensitive bacterium; *Micrococcus lysodeikticus* at the wavelength of 450 nm using the microplate ELISA reader. Immunoglobulin M (IgM) was assessed with an ELISA kit (Inova Biotechnology, China)^68^. Serum cortisol was determined by fish cortisol ELISA kit (Cusabio Biotech Co., LTD, China)^4^.

##### Histomorphological examination of liver and intestine

Liver and the whole intestine tissues of fish from different groups were used for histomorphological examination. The intestinal tissues (anterior, middle and posterior part) and liver samples (six samples/group) were collected and fixed in 10% formalin for 3 days. Then, the samples were dehydrated and washed multiple times in absolute alcohol before being embedded in paraffin. Serial 5-m longitudinal slices were cut on a Leica Rotary Microtome (RM 2145, Leica Microsystems) and placed on glass slides. The slides were then regularly stained with hematoxylin and eosin (H&E)^69^. The histomorphometric analysis of the intestine was performed using ImageJ analysis software (National Institutes of Health, MD, USA), whereas the intestinal villi length, width, and the inter-villi space were measured by ImageJ analysis software and expressed as µm.

##### Total RNA extraction, cDNA synthesis, and real-time quantitative PCR assay

Liver and brain samples (six samples/group) were collected in 2-mL sterile Eppendorf tubes from all tested groups. Then, the samples were immediately shocked in a liquid nitrogen. According to the manufacturer's instructions, total RNA was extracted from 50 mg of tissue using genazole (iNtRON Biotechnology).The integrity of extracted RNA was confirmed by ethidium bromide-stained 2% agarose gel electrophoresis. NanoDrop® BioDrop Spectrophotometer assessed RNA concentration. Following the manufacturer's instructions, 5 μg of extracted RNA sample was reverse transcribed using EasyScript First-strand cDNA synthesis Supermix (Oligo dT primer) (TransGenBiotechnology, China). Gene expression analysis was performed in the Rotor-Gene Q Real-time PCR system^70^ using the TOPreal qPCR 2 × PreMIX (enzynomics, Korea) using goldfish gene-specific primers (Table 1) with β-actin as a normalizer (housekeeping) gene. The results were analyzed by Rotor-Gene Q series software 2.3.3; relative expression of selected genes was calculated by 2^−ΔΔCT^ method according to^71^.

**Table 1:** Primers used for gene expression analysis.

Gene	Primer sequence 5ʹ–3ʹ	NCBI accession number	References
β-Actin forward β-Actin reverse	GATGATGAAATTGCCGCACTG ACCGACCATGACGCCCTGATGT	AB039726	Lu et al. (2013)^118^
GH forward GH reverse	CCCTCTGTCTTTCTGCAATTCTG GCGGAAAGAAATGCGAAGAAG	EU157192	Yang et al. (2021)^117^
SL-α forward SL-α reverse	GGAATCAGGGAGGAACCATGT ACCGAGTGAAGCAGCCATTT	EU580712	Yang et al. (2021)^117^
SL-β forward SL-β reverse	AACGGTGTCGGTTCCTATGTCT CGCCTGTACATCTACCAGTGGAT	U72940	Yang et al. (2021)^117^

Growth hormone (GH), Somatolactin α (SL-α), Somatolactin β (SL-β), β-actin (housekeeping gene).

### Statistical analysis

After data validation for normality and homogeneity, statistical analysis was performed using GraphPad Prism 6.01 software. The data was analyzed using one-way analysis of variance (ANOVA) and Tukey's post hoc test to find out any significant differences between the tested groups when the *P*-value < 0.05. Means and standard error of the mean (SEM) were used to express the findings.

### Study approval

The study is reported in accordance with Animal Research: Reporting of In Vivo Experiments (ARRIVE) guidelines.

## Results

### Artemia

#### Proximate composition and characteristics of Artemia after 24 h-enrichment process

Table 2 displays the performance of *Artemia* after 24 h of enrichment, including survival (%), individual length (µm), total *Artemia* biomass (g L^−1^), and population density (numbers L^−1^). There were significant (*P* < 0.05) differences in the survival rate and biomass among *Artemia* groups. Low survival was recorded in SEA compared to the other *Artemia* groups. However, the largest total *Artemia* biomass production (g L^−1^) was recorded in SEA group. Moreover, there was a significant (*P* < 0.05) difference among experimental groups in population density and the lowest record was presented in the SEA. Non-significant (*P* > 0.05) differences were recorded in individual length of *Artemia* (µm) among groups.

**Table 2:** Performance of *Artemia* (mean ± SEM) after 24 h enrichment process.

Parameter	Type of *Artemia*				*P*-value
	UEA	SEA	CEA	SCA	
Survival (%)	96.39 ± 1.085^a^	81.71 ± 0.905^b^	98.02 ± 0.405^a^	97.34 ± 0.473^a^	0.0001
Individual length (µm)	591.4 ± 31.61	607.7 ± 28.00	601.2 ± 22.82	632.7 ± 52.56	0.8623
Total *Artemia* biomass (g L^−1^)	0.444 ± 0.005^c^	0.525 ± 0.006^a^	0.468 ± 0.005^b^	0.491 ± 0.004^b^	0.0001
Population density (numbers L^−1^)	188,000 ± 2082^a^	159,333 ± 1764^b^	191,143 ± 790^a^	189,814 ± 922^a^	0.0001

UEA = Un-enriched *Artemia*; SEA = Spirulina-enriched *Artemia*; CEA = Canthaxanthin-enriched *Artemia*; SCA = Spirulina & Canthaxanthin-enriched *Artemia.*

Means within the same row lack common superscripts are significantly different (*P* < 0.05).

The proximate chemical composition of the experimental *Artemia* (UEA, SEA, CEA, and SCA) is presented in Table 3. The SCA exhibited the highest levels of protein and total energy, and the lowest moisture content compared to the other groups of *Artemia*. While, SEA had the highest lipid content and fiber content compared to the other groups of *Artemia*.

**Table 3:** Proximate chemical composition (means ± SEM) of *Artemia* used for feeding experimental goldfish based on DM basis.

Parameter	Type of Artemia				*P*-value
	UEA	SEA	CEA	SCA	
Crude protein (%)	46.37 ± 0.15^d^	47.23 ± 0.13^c^	49.32 ± 0.015^b^	50.12 ± 0.06^a^	0.0001
Crude lipid (%)	7.35 ± 0.03^b^	8.29 ± 0.14^a^	7.75 ± 0.02^ab^	7.59 ± 0.21^b^	0.0044
Ash (%)	10.54 ± 0.05^a^	9.02 ± 0.01^b^	10.20 ± 0.14^a^	10.50 ± 0.17^a^	0.0001
Fiber (%)	3.07 ± 0.01^a^	3.13 ± 0.09^a^	2.85 ± 0.02^ab^	2.70 ± 0.09^b^	0.0051
Moisture (%)	17.21 ± 0.02^a^	13.70 ± 0.09^b^	12.55 ± 0.03^c^	10.80 ± 0.15^d^	0.0001
Total energy (Kcal kg^−1^)	3578 ± 20.90^c^	3633 ± 14.70^c^	3715 ± 8.60^b^	3801 ± 1.60^a^	0.0001

UEA = Un-enriched *Artemia*; SEA = Spirulina-enriched *Artemia*; CEA = Canthaxanthin-enriched *Artemia*; SCA = Spirulina & Canthaxanthin -enriched *Artemia.*

Means within the same row lack common superscripts are significantly different (*P* < 0.05).

#### Total carotenoids of Artemia

**Figure 2:**
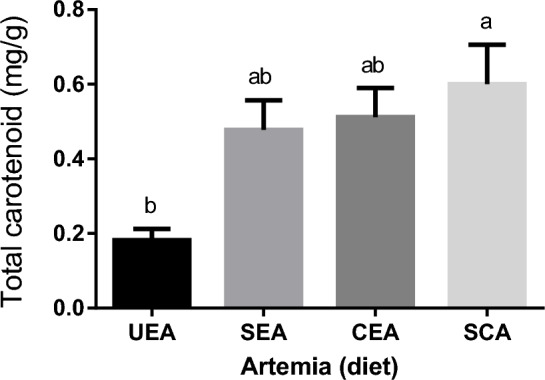
Total carotenoid content (means ± SEM) of *Artemia* used for feeding experimental goldfish. Means within the same column lack common superscripts are significantly different (*P* < 0.05). UEA = Un-enriched *Artemia*; SEA = Spirulina-enriched *Artemia*; CEA = Canthaxanthin-enriched *Artemia*; SCA = Spirulina & Canthaxanthin -enriched *Artemia*.

Figure 2 illustrates the total carotenoid content of un-enriched and enriched *Artemia* with spirulina and/or canthaxanthin used for feeding goldfish. Data showed significant differences (*P* < 0.05) among *Artemia* groups with the highest carotene content being detected in *Artemia* enriched with spirulina and canthaxanthin (SCA) followed by CEA and SEA. However, the lowest carotenoid content was detected in the UEA.

#### GC–MS fatty acid profile of un-enriched and carotenoid-enriched Artemia

Fatty acid profiles (% of total fatty acids) of un-enriched and enriched *Artemia* with spirulina, and/or canthaxanthin used for feeding goldfish are presented in Table 4. The results indicated that the SCA group had more polyunsaturated fatty acids (PUFA) as linolenic acid (C18:3 n-3), unsaturated fatty acids (UFA), docosahexaenoic acid (DHA), and omega3 (ω3) fatty acids than the other tested groups. The UEA had more saturated fatty acids (SFA) as palmitic acid (C16:0), monounsaturated fatty acids (MUFA) as Oleic acid (C18:1 n-9), and omega6 (ω6) fatty acids as Ecosatrienoic acid (C20:3 n-6) than the enriched *Artemia* groups. SEA and CEA demonstrated comparable effects.

**Table 4:** Fatty acid profile (% of total fatty acids) of *Artemia* used for feeding experimental goldfish.

Fatty acid	Type of *Artemia*			
	UEA	SEA	CEA	SCA
C9:0	0.09	0.15	nd	0.09
C14:0	1.64	1.65	1.45	1.73
C16:0	16.25	11.86	11.83	9.55
C17:0	1.03	1.25	1.07	1.53
C18:0	5.12	6.92	6.25	5.97
C16:1	3.78	3.94	3.61	3.09
C17:1	0.74	0.86	0.71	0.88
C18:1 (n-9)	25.36	22.39	24.92	19.06
C18:2 (n-3)	5.86	6.1	5.78	7
C18:3(n-6)	0.95	0.38	0.71	0.49
C18:3 (n-3)	20.32	24.07	nd	35.14
C18:3 (n-3) C	nd	nd	25.6	nd
C20:1	nd	nd	0.05	nd
C20:3 (n-6)	2.14	0.1	0.1	0.51
C22:4 (n-6)	nd	nd	nd	0.26
C20:4 (n-6)	0.68	1.1	1.03	0.07
C20:5 (n-3)	1.32	1.57	1.43	3.17
ƩSFA	24.13	21.83	20.6	18.87
ƩMUFA	29.88	27.19	29.29	23.03
ƩPUFA	31.27	33.32	34.65	46.64
ƩUFA	61.15	60.51	63.94	69.67
PUFA/SFA	1.295	1.526	1.682	2.471
Ʃ(n-3)	27.5	31.74	32.81	45.31
Ʃ(n-6)	3.77	1.58	1.84	1.33
n3/n6	0.137	0.049	0.056	0.293
EPA/DHA	1.32	1.57	1.43	3.17
Unidentified	14.73	15.06	13.16	11.59

UEA = Un-enriched *Artemia*; SEA = Spirulina-enriched *Artemia*; CEA = Canthaxanthin-enriched *Artemia*; SCA = Spirulina & Canthaxanthin-enriched *Artemia, *nd = Not detected.

### Goldfish

#### Water quality parameters

Water quality parameters of goldfish aquariums are illustrated in Table 5. There were no significant (*P* > 0.05) differences among all treatments in terms of pH, temperature, dissolved oxygen (DO), total ammonia nitrogen (TAN), unionized ammonia (UIA), electric conductivity (EC), and total dissolved salts (TDS).

**Table 5:** Water quality parameters (means ± SEM) of goldfish fed un-enriched and enriched *Artemia* with spirulina and/or canthaxanthin for 90 days.

Parameter	Experimental treatment					*P*-value
	T1	T2	T3	T4	T5	
pH	7.61 ± 0.16	7.48 ± 0.18	7.52 ± 0.21	7.74 ± 0.19	7.82 ± 0.09	0.6041
Temperature (°C)	28.43 ± 0.27	28.73 ± 0.07	28.30 ± 0.35	27.67 ± 0.34	28.47 ± 0.34	0.1937
DO (ppm)	6.10 ± 0.06	6.21 ± 0.10	6.24 ± 0.13	6.25 ± 0.14	6.28 ± 0.07	0.7892
TAN (ppm)	0.25 ± 0.04	0.28 ± 0.03	0.29 ± 0.012	0.27 ± 0.02	0.24 ± 0.02	0.6333
UIA (ppm)	0.0073 ± 0.002	0.0074 ± 0.003	0.0081 ± 0.003	0.0109 ± 0.004	0.0108 ± 0.001	0.8120
EC (µS)	468.1 ± 5.15	468.8 ± 9.98	472.3 ± 3.88	473.7 ± 2.89	474.3 ± 6.80	0.9304
TDS (ppm)	298.1 ± 36.18	350.6 ± 6.06	302.0 ± 42.37	300.8 ± 41.68	301.4 ± 41.82	0.8192

T1 = goldfish fed commercial diet; T2 = goldfish fed commercial diet and un-enriched *Artemia*; T3 = goldfish fed commercial diet and spirulina-enriched *Artemia*; T4 = goldfish fed commercial diet and canthaxanthin-enriched *Artemia*; T5 = goldfish fed commercial diet and spirulina and canthaxanthin- enriched *Artemia.*

*DO* Dissolved oxygen, *TAN* total ammonia nitrogen, *UIA* unionized ammonia, *EC* electric conductivity, *TDS* total dissolved salts.

#### Growth performance and condition factor

Results of growth performance are illustrated in Table 6. There were significant (*P* < 0.05) differences among treatments in terms of final weight, weight gain, average daily gain (ADG), specific growth rate (SGR), final length, length gain and condition factor (K). Fish fed spirulina and canthaxanthin-enriched *Artemia* (T5) showed the highest values of growth performance, and lowest values of condition factor.

**Table 6:** Growth performance (means ± SEM) of goldfish fed un-enriched and enriched *Artemia* with spirulina and/or canthaxanthin for 90 days.

Parameter	Experimental treatment					*P*-value
	T1	T2	T3	T4	T5	
Final weight^1^ (g)	4.150 ± 0.145^b^	4.340 ± 0.179^b^	4.410 ± 0.141^b^	4.437 ± 0.117^ab^	5.030 ± 0.042^a^	0.0090
Weight gain (g)	3.057 ± 0.157^b^	3.247 ± 0.194^b^	3.317 ± 0.147^ab^	3.327 ± 0.130^ab^	3.930 ± 0.049^a^	0.0155
ADG (g fish^−1^ day^−1^)	0.034 ± 0.003^b^	0.036 ± 0.00^b^	0.037 ± 0.002^ab^	0.037 ± 0.001^ab^	0.044 ± 0.001^a^	0.0153
SGR (% day^−1^)	1.482 ± 0.048	1.530 ± 0.062	1.547 ± 0.044	1.539 ± 0.043	1.689 ± 0.020	0.0773
Survival (%)	93.33 ± 3.85	95.56 ± 4.44	97.78 ± 2.223	100.0 ± 0.0	100.0 ± 0.0	0.4239
Final length^2^ (cm)	4.67 ± 0.16^c^	5.28 ± 0.36^bc^	5.67 ± 0.33^bc^	6.17 ± 0.45^b^	7.69 ± 0.16^a^	0.0005
Length gain (cm fish^−1^)	1.66 ± 0.17^c^	2.27 ± 0.36^bc^	2.66 ± 0.33^bc^	3.16 ± 0.44^b^	4.68 ± 0.16^a^	0.0005
Condition factor (K)	4.137 ± 0.487^a^	3.053 ± 0.451^ab^	2.570 ± 0.590^ab^	2.013 ± 0.426^b^	1.113 ± 0.072^b^	0.0074

T1 = goldfish fed commercial diet only; T2 = goldfish fed commercial diet and un-enriched *Artemia*; T3 = goldfish fed commercial diet and spirulina-enriched *Artemia*; T4 = goldfish fed commercial diet and canthaxanthin-enriched *Artemia*; and T5 = goldfish fed commercial diet and spirulina and canthaxanthin- enriched *Artemia.*

1: Initial weight (gm) = 1.093 ± 0.012.

2: Initial length (cm) = 3.01 ± 0.01, ADG = Average daily gain, SGR = Specific growth rate (SGR).

Means within the same row lack common superscripts are significantly different (*P* < 0.05).

#### Proximate chemical composition

Table 7 illustrates the approximate chemical composition of goldfish fed unenriched and enriched *Artemia* with spirulina and/or canthaxanthin. Dry matter, moisture, crude protein, crude fat, fiber, ash, carbohydrate, and energy varied significantly (*P* < 0.05) among experimental goldfish. The goldfish fed spirulina and canthaxanthin-enriched *Artemia* (T5) had the highest protein levels, however the goldfish fed commercial diet and spirulina-enriched *Artemia* (T3) group had the highest carcass energy levels. Moreover, the highest crude fat level was reported in T2 and T4, and the highest carbohydrate level was recorded in T1.

**Table 7:** Proximate chemical composition on dry matter basis (means ± SEM) of goldfish fed un-enriched and enriched *Artemia* with spirulina and/or canthaxanthin for 90 days.

Parameters	Experimental treatment					*P*-value
	T1	T2	T3	T4	T5	
Crude protein (%)	52.33 ± 0.06^e^	52.63 ± 0.02^d^	53.93 ± 0.03^c^	54.35 ± 0.06^b^	54.75 ± 0.06^a^	0.0001
Crude lipid (%)	24.14 ± 0.01^a^	24.16 ± 0.04^a^	23.45 ± 0.01^b^	24.16 ± 0.01^a^	23.46 ± 0.03^b^	0.0001
Ash %	18.23 ± 0.06^b^	18.65 ± 0.02^a^	18.34 ± 0.01^b^	16.94 ± 0.05^d^	17.26 ± 0.02^c^	0.0001
Fiber (%)	1.78 ± 0.10^c^	1.94 ± 0.02^bc^	2.25 ± 0.01^a^	1.850 ± 0.04^bc^	2.04 ± 0.03^ab^	0.0001
Carbohydrate (%)	5.27 ± 0.03^a^	4.50 ± 0.02^b^	4.26 ± 0.01^c^	4.68 ± 0.11^b^	4.52 ± 0.01^b^	0.0001
Moisture (%)	73.67 ± 0.08^c^	74.59 ± 0.17^ab^	75.01 ± 0.01^a^	72.81 ± 0.08^d^	74.37 ± 0.10^b^	0.0001
Carcass energy (Kcal 100g^−1^)	525.0 ± 0.26^d^	525.6 ± 0.23^d^	534.6 ± 0.19^a^	530.3 ± 0.62^b^	523.0 ± 0.34^c^	0.0001

T1 = goldfish fed commercial diet; T2 = goldfish fed commercial diet and un-enriched *Artemia*; T3 = goldfish fed commercial diet and spirulina-enriched *Artemia*; T4 = goldfish fed commercial diet and canthaxanthin-enriched *Artemia*; T5 = goldfish fed commercial diet and spirulina and canthaxanthin- enriched *Artemia.*

Means within the same row lack common superscripts are significantly different (*P* < 0.05).

#### Concentration of β-carotene

**Figure 3:**
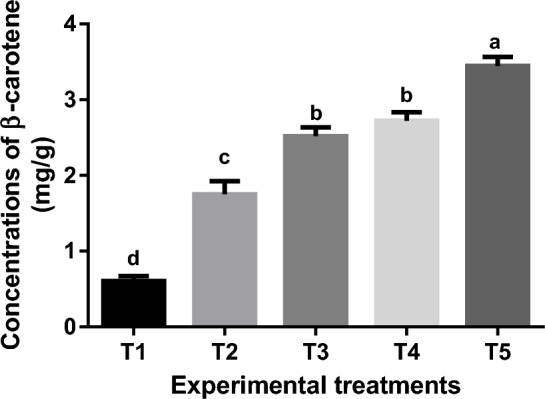
Mean concentrations of β-carotene (mg g^−1^) of experimental goldfish extracted and measured using high-performance liquid chromatography (HPLC). Means within the same column lack common superscripts are significantly different (*P* < 0.05). T1 = goldfish fed commercial diet; T2 = goldfish fed commercial diet and un-enriched *Artemia*; T3 = goldfish fed commercial diet and spirulina-enriched *Artemia*; T4 = goldfish fed commercial diet and canthaxanthin-enriched *Artemia*; T5 = goldfish fed commercial diet and spirulina and canthaxanthin- enriched *Artemia.*

Figure 3 represents the mean β-carotene concentrations (mg g^−1^) of goldfish fed commercial diet, unenriched *Artemia*, and *Artemia* enriched with spirulina and/or canthaxanthin. There was a significant (*P* < 0.05) difference between the experimental treatments in the concentration of β-carotene. The goldfish fed *Artemia*-supplemented diets exhibited greater (*P* < 0.05) β-carotene concentrations than control (T1) and unenriched *Artemia* group (T2). The highest (*P* < 0.05) β-carotene concentration was reported in T5 followed by T4 and T3 respectively.

#### Chromatic deformity and color intensity using computer-assisted image analysis

The assessment of chromatic deformities and color intensity in goldfish fed different feeding regimes are presented in Table 8. Significant differences (*P* < 0.05) in chromatic deformity among experimental treatments were detected. Goldfish fed commercial diet and spirulina and canthaxanthin-enriched *Artemia* (T5) had the lowest degree of chromatic deformities and a high level of color intensity and red index color. There were no significant differences (*P* > 0.05) between goldfish fed spirulina-enriched *Artemia* (T3) and canthaxanthin-enriched *Artemia* (T4).

**Table 8:** The assessment of chromatic deformities and color intensity (means ± SEM) of experimental goldfish using computer-assisted image analysis.

Parameter	Experimental treatment					*P*-value
	T1	T2	T3	T4	T5	
Chromatic deformity	81.34 ± 4.968^a^	76.11 ± 4.883^a^	53.45 ± 4.848^b^	49.04 ± 3.557^b^	40.09 ± 4.185^b^	0.0001
Color intensity^1^ (un-weighted)	115.4 ± 3.396^c^	122.9 ± 1.942^bc^	131.3 ± 2.634^bc^	133.7 ± 6.735^ab^	149.4 ± 4.726^a^	0.0001
Color intensity^2^ (weighted)	136.3 ± 14.24	125.7 ± 10.40	130.7 ± 13.97	104.7 ± 28.90	126.3 ± 11.57	0.7449
R + G + B	177.3 ± 1.667	177.3 ± 4.372	160.7 ± 13.86	151.0 ± 15.72	177.7 ± 7.446	0.2824
R	148.0 ± 17.69^b^	154.3 ± 9.821^b^	169.7 ± 13.37^ab^	195.3 ± 30.44^ab^	254.3 ± 19.63^a^	0.0189
G	130.0 ± 15.10	151.3 ± 12.47	127.3 ± 14.97	98.67 ± 30.69	122.7 ± 12.44	0.4338
B	97.33 ± 22.58	78.67 ± 16.25	108.7 ± 11.79	68.67 ± 29.46	85.67 ± 9.025	0.6349

T1 = goldfish fed commercial diet; T2 = goldfish fed commercial diet and un-enriched *Artemia*; T3 = goldfish fed commercial diet and spirulina-enriched *Artemia*; T4 = goldfish fed commercial diet and canthaxanthin-enriched *Artemia*; T5 = goldfish fed commercial diet and spirulina and canthaxanthin- enriched *Artemia.*

1: Intensity (un-weighted) = mode of R + G + B\3.

2: Intensity (weighted) = mode of 0.299R + 0.587G + 0.114B, Red + green + blue = mode of R + G + B, R, red; G, green; B, blue (grayscale values).

Means within the same row lack common superscripts are significantly different (*P* < 0.05).

#### Hematological parameters

Table 9 displays the haematological indices, including RBCs, Hb, PCV, WBCs, heterophil, lymphocytes and monocyte. There was an increase (*P* < 0.05) in RBCs, Hb, WBCs, and lymphocyte in goldfish fed the *Artemia* enriched diets (T3–T4–T5) compared to T2 and T1 with the highest values being reported in T5.

**Table 9:** Hematological analysis (means ± SEM) of goldfish fed un-enriched and enriched *Artemia* with spirulina and/or canthaxanthin for 90 days.

Parameter	Experimental treatment					*P*-value
	T1	T2	T3	T4	T5	
RBCs (× 10 mm^−3^)	2.72 ± 0.02^d^	2.80 ± 0.01^c^	2.93 ± 0.01^b^	2.97 ± 0.01^ab^	3.01 ± 0.00^a^	0.0001
Hb (g 100 ml^−1^)	8.38 ± 0.02^e^	8.45 ± 0.01^d^	8.91 ± 0.00^c^	9.05 ± 0.01^b^	9.13 ± 0.01^a^	0.0001
PCV (%)	26.50 ± 0.29	27.50 ± 0.29	28.00 ± 0.0	29.00 ± 0.0	29.00 ± 0.0	0.0801
WBCs (× 10^3^ mm^−3^)	17.42 ± 0.25^d^	18.62 ± 0.42^cd^	19.35 ± 0.38^bc^	20.01 ± 0.08^ab^	20.95 ± 0.23^a^	0.0001
Heterophil (× 10^3^ mm^−3^)	2.53 ± 0.01	2.50 ± 0.10	2.32 ± 0.07	2.40 ± 0.13	2.20 ± 0.04	0.0845
Lymphocyte (× 10^3^ mm^−3^)	13.07 ± 1.29^b^	14.17 ± 1.53^ab^	15.00 ± 1.35^a^	15.81 ± 1.07^a^	16.55 ± 1.18^a^	0.0022
Monocyte (× 10^3^ mm^−3^)	1.39 ± 0.08	1.58 ± 0.12	1.74 ± 0.03	1.50 ± 0.15	1.89 ± 0.12	0.0822

T1 = goldfish fed commercial diet; T2 = goldfish fed commercial diet and un-enriched *Artemia*; T3 = goldfish fed commercial diet and spirulina-enriched *Artemia*; T4 = goldfish fed commercial diet and canthaxanthin-enriched *Artemia*; T5 = goldfish fed commercial diet and spirulina and canthaxanthin-enriched *Artemia.*

*RBCs* red blood cells, *HB* hemoglobin, *PCV* packed cell volume, *WBCs* white blood cells.

Means within the same row lack common superscripts are significantly different (*P* < 0.05).

#### Serum biochemical profile and digestive enzymes activity

**Figure 4:**
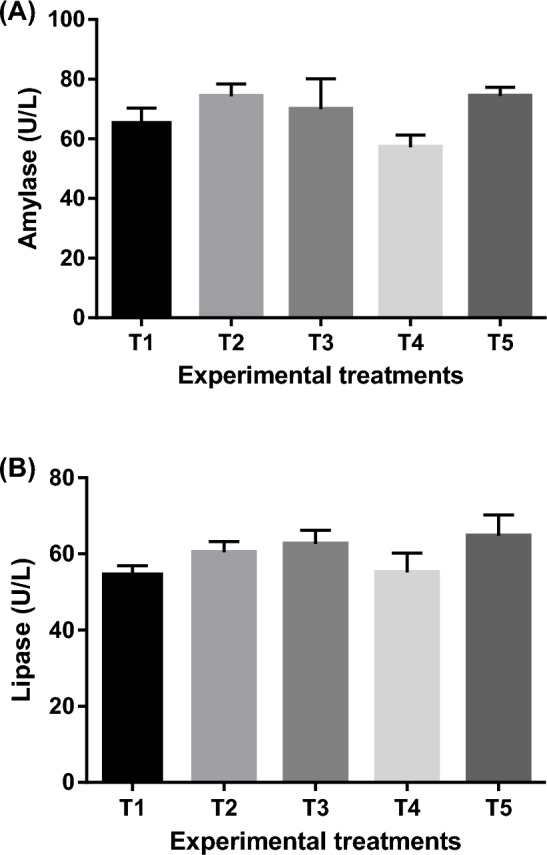
Means ± SEM of digestive enzyme activity [Amylase activity (**A**) and Lipase activity (**B**)] of experimental goldfish fed un-enriched and enriched *Artemia* with spirulina and/or canthaxanthin for 90 days. Means within the same column lack common superscripts are significantly different (*P* < 0.05). T1 = goldfish fed commercial diet only; T2 = goldfish fed commercial diet and un-enriched *Artemia*; T3 = goldfish fed commercial diet and spirulina-enriched *Artemia*; T4 = goldfish fed commercial diet and canthaxanthin-enriched *Artemia*; and T5 = goldfish fed commercial diet and spirulina and canthaxanthin- enriched *Artemia.*

Table 10 illustrates the serum biochemical analysis. The values of ALT, AST, cholesterol, triglycerides and glucose showed non-significant (*P* > 0.05) differences amongst experimental treatments. There were however, significant (*P* < 0.05) differences between experimental treatments in total protein, albumin and globulin with the highest values being observed in T5 and T4. The digestive enzyme activity (amylase, and lipase) presented in Fig. 4 showed no significant (*P* > 0.05) differences between experimental treatments.

**Table 10:** Serum biochemical analysis (means ± SEM) of goldfish fed un-enriched and enriched *Artemia* with spirulina and/or canthaxanthin for 90 days.

Parameter	Experimental treatment					*P*-value
	T1	T2	T3	T4	T5	
ALT (U l^−1^)	20.26 ± 1.09	19.41 ± 0.42	19.00 ± 0.79	17.05 ± 0.98	17.51 ± 0.30	0.0758
AST (U l^−1^)	17.74 ± 0.22	17.11 ± 0.12	17.07 ± 0.28	16.57 ± 0.34	17.25 ± 0.44	0.1857
Total protein (g dl^−1^)	3.59 ± 0.01^c^	3.71 ± 0.01^b^	3.75 ± 0.03^b^	3.89 ± 0.01^a^	3.89 ± 0.01^a^	0.0001
Albumin (g dl^−1^)	1.25 ± 0.01^c^	1.27 ± 0.01^c^	1.33 ± 0.01^b^	1.38 ± 0.01^a^	1.38 ± 0.01^a^	0.0001
Globulin (g dl^−1^)	2.34 ± 0.02^b^	2.44 ± 0.01^ab^	2.42 ± 0.04^ab^	2.51 ± 0.0^1a^	2.51 ± 0.01^a^	0.0014
Triglyceride (mg dl^−1^)	80.81 ± 0.32	82.06 ± 1.27	81.82 ± 0.94	79.55 ± 0.18	83.56 ± 1.93	0.2142
Cholesterol (mg dl^−1^)	70.02 ± 0.15	70.82 ± 0.30	71.19 ± 1.13	68.54 ± 0.95	71.22 ± 0.48	0.1103
Glucose (mg dl^−1^)	9.80 ± 0.20	11.10 ± 0.14	10.85 ± 0.28	11.08 ± 0.28	11.12 ± 0.71	0.1429

T1 = goldfish fed commercial diet; T2 = goldfish fed commercial diet and un-enriched *Artemia*; T3 = goldfish fed commercial diet and spirulina-enriched *Artemia*; T4 = goldfish fed commercial diet and canthaxanthin-enriched *Artemia*; T5 = goldfish fed commercial diet and spirulina and canthaxanthin- enriched *Artemia.*

*ALT* alanine aminotransferase, *AST* aspartate aminotransferase.

Means within the same row lack common superscripts are significantly different (*P* < 0.05).

#### Immune response parameters

**Figure 5:**
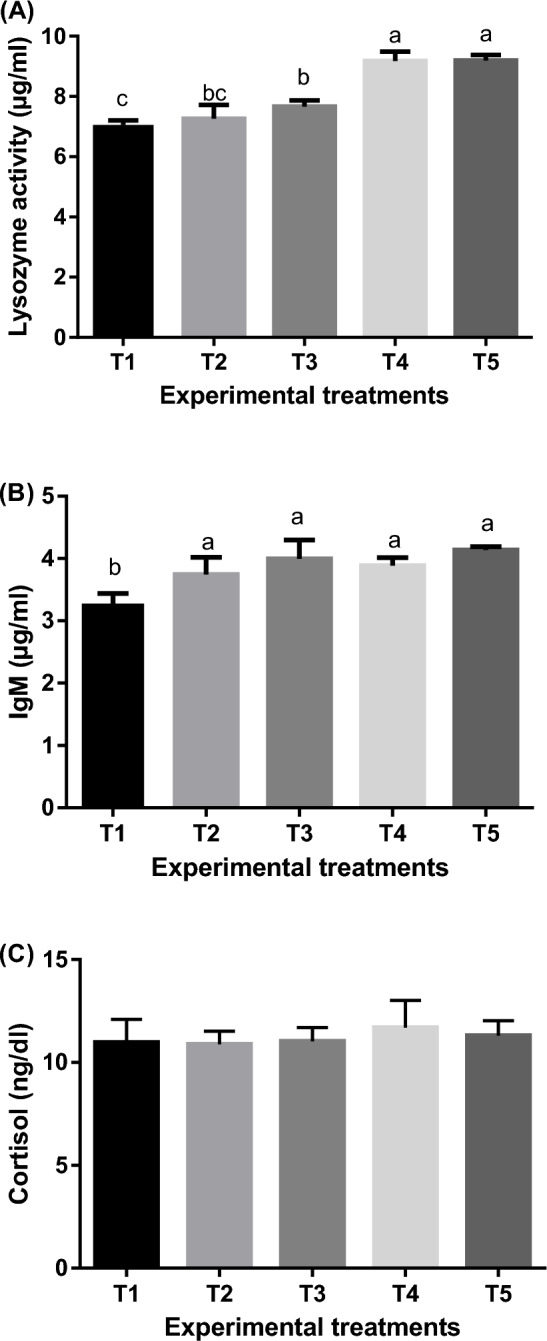
Means ± SEM of immune response [Lysozyme activity (**A**), immunoglobulin M, IgM (**B**) and cortisol (**C**)] of experimental goldfish fed un-enriched and enriched *Artemia* with spirulina and/or canthaxanthin for 90 days. Means within the column lack common superscripts are significantly different (*P* < 0.05). T1 = goldfish fed commercial diet; T2 = goldfish fed commercial diet and un-enriched *Artemia*; T3 = goldfish fed commercial diet and spirulina-enriched *Artemia*; T4 = goldfish fed commercial diet and canthaxanthin-enriched *Artemia*; T5 = goldfish fed commercial diet and spirulina and canthaxanthin- enriched *Artemia.*

Figure 5 displays the immune response [Lysozyme activity, immunoglobulin M, (IgM) and cortisol] of the experimental goldfish fed un-enriched and enriched *Artemia* with spirulina and/or canthaxanthin. There were variations (*P* < 0.05) in lysozyme activity and IgM levels between experimental treatments, with the highest values being observed in T5. However, there was no significant (*P* > 0.05) difference between experimental treatments regarding levels of cortisol.

#### Histomorphology of the intestine and liver

**Figure 6:**
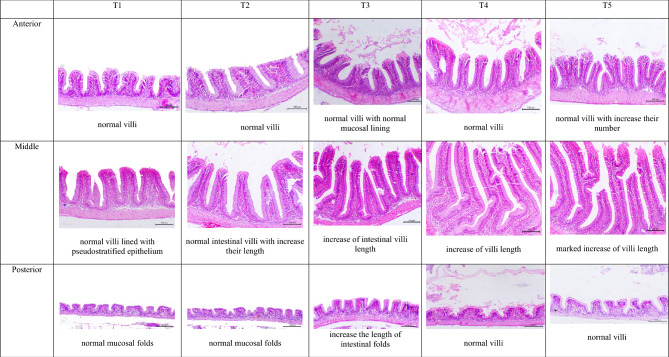
Photomicrograph shows the anterior, middle, and posterior portions of the intestine of experimental goldfish fed un-enriched and enriched *Artemia* with spirulina and/or canthaxanthin for 90 days, stained with Hematoxylin and Eosin (H&E), X200, bar = 100 µm. T1 = goldfish fed commercial diet; T2 = goldfish fed commercial diet and un-enriched *Artemia*; T3 = goldfish fed commercial diet and spirulina-enriched *Artemia*; T4 = goldfish fed commercial diet and canthaxanthin-enriched *Artemia*; T5 = goldfish fed commercial diet and spirulina and canthaxanthin- enriched *Artemia.*

**Figure 7:**
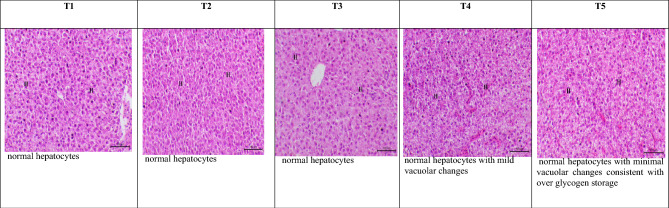
Photomicrograph shows the liver of experimental goldfish fed un-enriched and enriched *Artemia* with spirulina and/or canthaxanthin for 90 days showing normal hepatocytes (H letters indicate hepatocytes), (H&E), X200, bar = 100 µm. T1 = goldfish fed commercial diet; T2 = goldfish fed commercial diet and un-enriched *Artemia*; T3 = goldfish fed commercial diet and spirulina-enriched *Artemia*; T4 = goldfish fed commercial diet and canthaxanthin-enriched *Artemia*; T5 = goldfish fed commercial diet and spirulina and canthaxanthin- enriched *Artemia.*

The morphometric analysis of intestinal tissues showed significant (*P* < 0.05) differences between experimental treatments (Table 11 and Fig. 6) in the different sections of intestine (anterior, middle and posterior). Villi length and goblet cells number in the anterior and middle portions of intestine and villi width and goblet cells number in the posterior portion of intestine showed significant (*P* < 0.05) differences between experimental treatments with the highest values being reported in T5. The histological investigations of liver tissue showed a similar anatomical arrangement with normal structure in the experimental treatments. Hepatocytes appeared polyhedral in shape, with nuclei usually spherical, big, and centered in the cell. The nucleolus was visible, and there were granules in the cytoplasm. The liver parenchyma, the cells were unevenly dispersed and divided by sinusoidal capillaries. Small blood arteries, bile ducts, and an exocrine pancreas with serous acinar cells with eosinophilic staining at the apical region and basophilic staining towards the base were also seen (Fig. 7).

**Table 11:** Intestinal morphometric analysis (means ± SEM) of goldfish fed un-enriched and enriched *Artemia* with spirulina and/or canthaxanthin for 90 days.

Intestinal portion	Variable	Experimental treatment					*P*-value
		T1	T2	T3	T4	T5	
Anterior	Villi length (μm)	101.1 ± 13.31^c^	139.3 ± 8.67^bc^	178.1 ± 6.00^ab^	181.7 ± 9.06^a^	188.2 ± 4.87^a^	0.0002
	Villi width (μm)	76.27 ± 4.49	74.59 ± 11.34	57.89 ± 4.92	57.98 ± 6.28	66.58 ± 3.63	0.2248
	Inter villi space (μm)	42.17 ± 7.73	52.40 ± 4.62	39.98 ± 3.43	48.07 ± 13.45	55.31 ± 4.82	0.5994
	Goblet cells (# mm^−2^)	9.67 ± 0.67^b^	10.67 ± 1.20^b^	17.33 ± 0.88^a^	16.33 ± 1.20^a^	20.67 ± 0.88^a^	0.0001
Middle	Villi length (μm)	190.2 ± 4.03^c^	254.5 ± 19.73^b^	310.7 ± 0.56^b^	421.2 ± 11.00^a^	442.4 ± 18.41^a^	0.0001
	Villi width (μm)	61.61 ± 7.21	66.31 ± 5.08	79.68 ± 3.67	75.24 ± 8.85	70.36 ± 1.03	0.2777
	Inter villi space (μm)	40.79 ± 2.42	42.78 ± 2.78	54.12 ± 11.52	30.46 ± 0.63	47.80 ± 14.04	0.3992
	Goblet cells (# mm^–2^)	16.00 ± 0.58^d^	18.33 ± 0.88^cd^	21.00 ± 1.16^bc^	23.33 ± 0.88^b^	30.33 ± 0.88^a^	0.0001
Posterior	Villi length (μm)	59.03 ± 3.36	69.47 ± 6.75	52.11 ± 20.39	114.6 ± 10.25	71.84 ± 20.11	0.0712
	Villi width (μm)	61.36 ± 1.62^b^	58.37 ± 1.41^b^	85.35 ± 3.56^ab^	82.07 ± 7.63^ab^	100.4 ± 11.96^a^	0.0058
	Inter villi space (μm)	54.67 ± 9.40	53.62 ± 6.02	61.34 ± 3.76	71.54 ± 5.55	74.71 ± 16.08	0.4164
	Goblet cells (# mm^−2^)	5.33 ± 0.33^b^	6.67 ± 0.33^b^	10.00 ± 0.58^a^	9.67 ± 0.33^a^	11.00 ± 0.58^a^	0.0001

T1 = goldfish fed commercial diet; T2 = goldfish fed commercial diet and un-enriched *Artemia*; T3 = goldfish fed commercial diet and spirulina-enriched *Artemia*; T4 = goldfish fed commercial diet and canthaxanthin-enriched *Artemia*; T5 = goldfish fed commercial diet and spirulina and canthaxanthin- enriched *Artemia.*

Means within the same row lack common superscripts are significantly different (*P* < 0.05).

#### Expression of growth hormone, somatolactin-β and somatolactin-α genes

**Figure 8:**
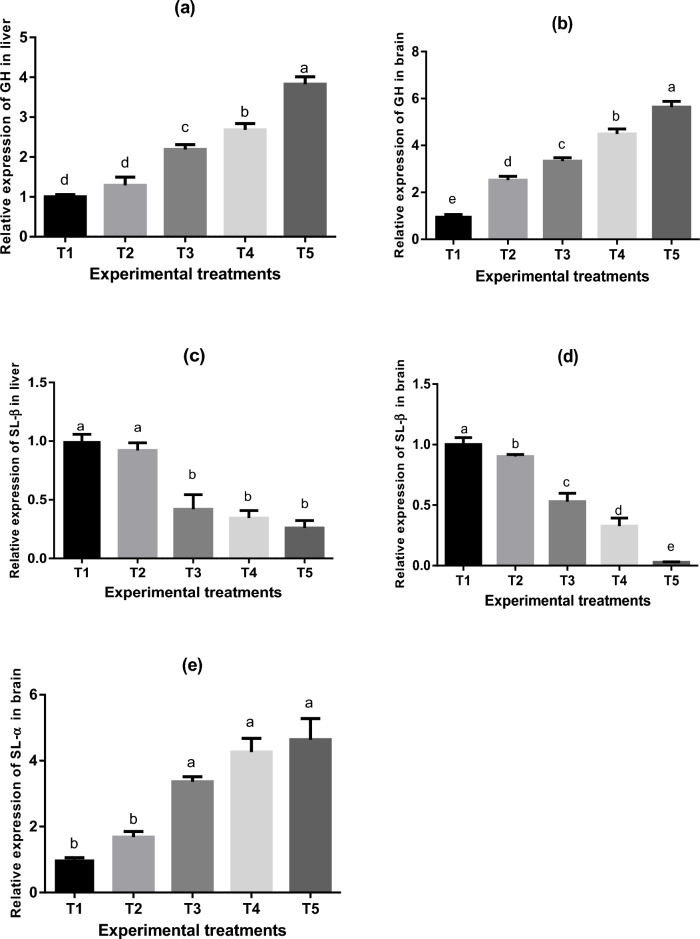
Means ± SEM of Gene expressions [GH = growth hormone in liver and brain (**a**) and (**b**), SL-β = somatolactin β in liver and brain (**c**) and (**d**), SL-α = somatolactin α in brain (**e**)] of experimental goldfish fed un-enriched and enriched *Artemia* with spirulina and/or canthaxanthin for 90 days. Means within the same column lack common superscripts are significantly different (*P* < 0.05). T1 = goldfish fed commercial diet; T2 = goldfish fed commercial diet and un-enriched *Artemia*; T3 = goldfish fed commercial diet and spirulina-enriched *Artemia*; T4 = goldfish fed commercial diet and canthaxanthin-enriched *Artemia*; T5 = goldfish fed commercial diet and spirulina and canthaxanthin- enriched *Artemia.*

Fold changes of growth hormone, somatolactin β and somatolactin-α genes in the goldfish brain and liver fed un-enriched and enriched *Artemia* with spirulina and/or canthaxanthin were demonstrated in Fig. 8. There were variations (*P* < 0.05) in SL-α and GH gene expression among experimental treatments. In goldfish fed *Artemia*, there was an increase in GH gene expression in the liver and brain, with the greatest values being observed in T5. However, a decrease in SL-β gene expression was observed in the liver and brain (T5 < T4 < T3 < T2 < T1). SL-α gene expression was detected in the brain with higher expression in *Artemia*-enriched treatments, particularly in T5.

## Discussion

The enrichment of brine shrimp, particularly during larval rearing, makes *Artemia* crucial to manage goldfish hatcheries. The main objective of *Artemia* enrichment is to improve *Artemia* nutritional value that has a significant effect on the quality of the fish larvae.

In the present study, the performance of *Artemia* after 24 h enrichment process revealed that the use of canthaxanthin powder improved the survival, and population density of canthaxanthin-enriched *Artemia* (CEA). However, spirulina improved total *Artemia* biomass of spirulina-enriched *Artemia* (SEA). Similar results were reported by^72,73^. Moreover, Ref.^74^ found that feeding Artima on high β-carotene algae improved their performance. This may be due to *Artemia* biomass output being greatly influenced by the quantity and quality of food available, as well as the rising environment^75^. Moreover, canthaxanthin powder, used to enrich *Artemia* as an artificial source of carotenoids, can be easily dissolved in water with a smaller particle size than spirulina^72,76^. This may provide *Artemia* with its nutritional requirements. As a result, it is recommended to use canthaxanthin to improve *Artemia* survival rate and population density however; spirulina can be used to obtain higher biomass. Therefore, a mixture of canthaxanthin and spirulina appears to be a better choice in enriching brine shrimp.

This study examined the proximate chemical composition of un-enriched *Artemia* (UEA), SEA, CEA, and SCA. It was found that, SEA, CEA, and SCA improved the chemical composition of *Artemia* in the form of higher protein, fat and total energy content, lower moisture, fiber and ash compared to UEA. SCA had a high protein and energy content, although SEA had a high-fat level. This result is in line with the findings of^77^. This may be attributed to spirulina's high fat and protein levels^78^ and canthaxanthin's ability to motivate *Artemia* due to its high β-carotene content^79^. Therefore, canthaxanthin and/or spirulina can improve the chemical composition of brine shrimp.

In the current study, SCA had greater total β-carotene content than the other treatments. This may be due to the considerable pigmentation present in canthaxanthin and spirulina, which is naturally low in *Artemia*. The findings of this study agree with those reported by^74^ who stated that carotenoids, which are originally stored in algal cells, can be fed to *Artemia* and can have a great impact on its health and performance.

According to the current study, SCA treatment had a high concentration of total polyunsaturated fatty acids (ƩPUFA), total unsaturated fatty acids (ƩUFA), total omega 3 (ƩOmega 3), and Eicosapentaenoic acids (EPA), and a very low concentration of total saturated fatty acids (ƩSFA), and total high unsaturated fatty acids (ƩHUFA). Numerous studies have also demonstrated that *Artemia* is a rich source of polyunsaturated fatty acids (PUFA), which are necessary for the growth and metabolism of many aquatic species^80,81^. Furthermore, PUFA, such as Eicosapentaenoic acids (EPA) and Docosahexaenoic acids (DHA), have been shown to have a significant impact on the early growth development and survival rate of goldfish larvae^82,83^. Thus, canthaxanthin and/or spirulina can be effectively used as potent enrichment agents for improving *Artemia* growth performance, chemical composition, β-carotene content and polyunsaturated fatty acids concentration.

Maintaining optimal water conditions in a goldfish tank is crucial for effective keeping of goldfish in a tank and for improving its performance, especially during the larval rearing period, which is characterized by intense feeding with live-rich diets high in lipids and proteins. The water quality results of the current study revealed absence of any negative impacts of canthaxanthin and/or spirulina on goldfish water parameters with recorded values falling within the recommended ranges of goldfish. Similar water quality records were reported by^4^. This finding is compatible with that of^84^, who said that goldfish can be utilized to help maintain water quality in rearing tanks by eating algae and other residues such as lost feed. Goldfish can also be utilized to keep rearing tanks clean for longer periods of time^85^.

Goldfish growth rates rely entirely on the environment in which they are kept, as well as the amount of high-quality food they are fed. In the current study, feeding enriched *Artemia* to goldfish increased their growth rate, particularly in the T5 treatment. The findings of this study are consistent with those of other studies indicating that feeding goldfish natural live food resulted in better growth than feeding artificial feed^3,79^. Moreover, Ref.^86^ found that *Artemia* nauplii cannot meet the nutritional requirements for fish larval development when used as the only source of food due to the lack of essential polyunsaturated fatty acids.

The growth performance of goldfish was boosted by feeding them *Artemia* enriched with carotenoids, particularly *Artemia* supplemented with spirulina and canthaxanthin (T5) because of the high nutrition value of carotenoids, polyunsaturated fatty acids, unsaturated fatty acids (UFA), DHA, and omega3 (ω3) fatty acids present in spirulina and canthaxanthin-enriched *Artemia* (SCA) reported in the present study. Adult goldfish can grow on vegetable diets, however juvenile goldfish require a greater protein content in their feeds for better growth^87^. Furthermore, improved growth performance may be attributed to feeding goldfish live natural food rather than commercial diets which are dependent on the palatability, quality, and adequate quantity of feed, size, type, and physical appearance of the feed, as well as other factors such as attractiveness, feed acceptability, and assimilation of nutrients^3^. In the same context, Ref.^88^ reported the greatest length growth of goldfish larvae fed *Artemia* (15.8 mm), followed by those fed *Artemia* plus a commercial feed (14.8 mm) and those just fed a commercial feed (10.8 mm length). In contrast, there is debate over the contribution of carotenoids to fish growth promotion. There was no discernible improvement in *P. leopardus* growth performance following treatment with varying astaxanthin concentrations^89^.

Earlier studies found that fish given just a dry commercial feed had a poorer survival rate (61.9%) than those fed just *Artemia* (96.4%) or *Artemia* plus a commercial feed (94%). Reference^44^ stated that the growth and survival of goldfish larvae were better demonstrated by the biofloc culture method than by the clear water culture system. Regardless of the cultivation system, the higher supply level of *Artemia* nauplii (300 *Artemia* nauplii/fish larvae) resulted in the best growth of the goldfish larvae as compared to the lower densities of nauplii. Therefore, canthaxanthin and/or spirulina can be effectively used as powerful *Artemia* enrichment agents for improving goldfish growth performance and survival.

After 90 days of feeding, the chemical composition of goldfish indicated an increase in the protein content in response to feeding enriched *Artemia*. This may be due to the relatively higher amount of protein in enriched *Artemia* compared to either un-enriched or commercial feed as presented in this study. This finding is similar to that reported by^90^.

Due to their inability to biosynthesize carotenoids properly, ornamental fish, like other animals, require proper nutrition for the development of natural pigmentation and good health^91^. Fish with higher carotenoid contents and fewer chromatic deformities typically have more color improvement. In the current study, goldfish fed spirulina and canthaxanthin-enriched *Artemia* (T5) had the lowest value of chromatic deformity. Same patterns were observed by^92,93^. The distinct colors of goldfish compared to other fish may help to explain this. Red, green, and blue color intensity levels (Red–Green–Blue; RGB) seem to have a role in how redness becomes visible in goldfish. The unweighted color intensity and R values are enhanced in T5. The starting concentration of redness and genetic background are believed to control the rate at which the red color intensity changes, which might explain the observed variations in red color intensity among fish fed live food from various treatments. The results of the current investigation are consistent with those of^53,94^. Moreover, Ref.^95^ reported that feeding *Artemia franciscana* with *Dunaliella salina* rich in β-carotene improved platy fish (*Xiphophorus maculatus)* skin pigmentation and the skin color red index.

The increase in β-carotenoid content observed in this study when goldfish were fed spirulina and canthaxanthin-enriched *Artemia* (T5) as an exogenous source of carotenoid for three months was due to the fact that spirulina and canthaxanthin are both coloring agents^96–98^. Also, Ref.^95^ discovered that *Artemia franciscana* supplemented with β-carotene from one type of algae called *Dunaliella salina* increased the skin pigmentation, red index of the skin color, and total carotenoid concentration in platy fish. Similarly, the levels of carotenoids in the skin of *Astronotus ocellatus* that were given *Dunaliella salina*, a natural source of β-carotene, were substantially greater in comparison to those who were given commercial feed, as stated by^79^, who added that the accumulation of carotenoids in the skin stimulates fish color modulations. Therefore, feeding goldfish spirulina and/or canthaxanthin-enriched *Artemia* improved red index and pigmentation of the skin.

Several studies on the hematological and immunostimulant benefits of carotenoids on ornamental fish have been conducted^74,79,99^. The hematological parameters of goldfish fed spirulina and canthaxanthin-supplemented *Artemia* (T5) were enhanced. The goldfish's hematological levels in this study were within normal ranges^100,101^. All *Artemia*-enriched treatments are lower in ALT and AST levels compared to T1. This result is comparable to that of^102^. Reduced ALT and AST levels have been frequently associated with great improvements in fish health, particularly the liver^103^. In the current study, goldfish fed spirulina-canthaxanthin enriched*-Artemia* (T5) had a higher total protein, albumin, total immunoglobulin, and lysozyme activity compared to fish fed commercial diet (T1). The current study findings are consistent with the findings of^99^. Albumin and globulins are two types of serum proteins.

The liver produces albumin and an osmotic force that keeps the circulatory space at a constant fluid volume. Albumin serves as a protein transporter and a protein reserve^104^. Lysozyme is a component of a fish defense system that causes bacterial lysis as well as activation of the complement system and phagocytes^105^. Feeding goldfish on spirulina and canthaxanthin-supplemented *Artemia* (T5) induced a considerable increase in lysozyme activity and immunoglobulin M (IgM) levels in the present study. This is consistent with the finding of^106^, who found that while lysozyme activity in the 100, 200, and 400 mg kg^−1^ mixed herbal enriched meal groups did not differ significantly from the control group during the first week, it did during the second and fourth weeks. Immunoglobulins mediate adaptive humoral immunity in fish, with IgM form the bulk of immune responses in teleost fish^107^. In addition, Ref.^89^ concluded that dietary astaxanthin improved the antioxidant capacity, immunity and disease resistance of coral trout.

Carotene serves as a precursor to vitamin A, which is essential for immune system function, reduces free radicals, prevents lipid oxidation, preserves cell membrane flexibility, and reduces immunosuppressive peroxides Refs.^99,108^ found that β-carotene-enriched *Artemia* fed to fish improved non-specific immune responses in form of high total immunoglobulin, and lysozyme activity in comparison to the control group. Conclusively, feeding *Artemia* enriched with canthaxanthin and/or spirulina enhances the health status and immune response of goldfish.

The height and width of intestinal villi, as well as the number of goblet cells, directly affect the digestion and absorption of nutrients in fish^109^. In this study, histomorphometric examination of intestinal villi absorptive capacity revealed that T5 treatment showed an increase in villi length, villi width, and the number of goblet cells. The improvement in intestinal morphometry (height and width of intestinal villi, the number of goblet cells) correlates with our findings of increased goldfish growth performance. The improvement in the nutritional or environmental conditions of the fish results in an improvement in the digestive system, which is reflected in the growth improvement. In this regard, Ref.^110^ discovered that the Nile tilapia (*Oreochromis niloticus*) given *Quillaja saponaria* and/or *Yucca schidigera* had improved intestinal histomorphology (increase in intestinal villi length and width, decrease in the inter-villi gap, and increase in goblet cell count) compared to the control.

A histological examination of the liver of goldfish fed only a commercial diet (T1) revealed that the hepatocytes were properly arranged when compared to those of other fish species. While feeding goldfish on spirulina and canthaxanthin-supplemented *Artemia* (T5) resulted in minimal vacuolar changes and significant glycogen accumulation and feeding goldfish canthaxanthin-supplemented *Artemia* (T4) resulted in mild vacuolar changes. This finding is consistent with the results of ALT and AST of goldfish reported also in the present study. Similar results were reported by^111,112^. Thus, using of *Artemia* enriched with canthaxanthin and/or spirulina as a source of goldfish nutrition improved intestine and liver health and therefore enhanced feed absorption capacity and growth performance.

Goldfish are commonly used as model fish in research on hormones, diet, behavior, and stress^113^. In the pars intermedia of the pituitary gland, somatolactin (SL), a member of the GH family, is produced and related to lipid metabolism^114^. Additionally, by aggregating melanosomes, SL affects how teleosts' bodies are colored^115^. Compared to the T1, GH expression was elevated in the liver and brain of fish in all treatments of the current experiment. There is no SL-α expression in the livers of goldfish because SL-α expression is not detected elsewhere other than the brain^116^. SL-β expression is suppressed in both the brain and liver. However, SL-α is raised in the brain, as shown by^117^, who stated that SL-α and proopiomelanocortin (POMC), as well as SL-β and vertebrate melanin-concentrating hormone (Pmch1), are responsible for the darkening and lightening of body color in response to the background color. Members of the growth hormone (GH) family and somatolactin (SL) modulate energy metabolism, are also associated with teleost body color modulation because these hormones are involved in both the control of body color and energy metabolism. Reference^117^ investigated the effects of feeding status and background color on the expression of genes involved in the control of body color in goldfish and discovered that feeding status may influence the modulation of body color via SL-α and SL-β, however, these effects may be restricted in comparison to the effect of background color.

## Conclusion

The use of canthaxanthin and/or spirulina enhanced the protein percentage, β-carotene content, survival rate, and performance of *Artemia*. Goldfish that were fed enriched *Artemia* had improved immune-physiological, transcriptome, and pigmentation responses in addition to improved performance. When goldfish were fed on *Artemia* that was enhanced with both spirulina and canthaxanthin, the best profile of improved health and performance was achieved.

## Data Availability

All relevant data are available from the authors upon request.
